# Influence of Polymer Inclusion Membrane Composition on Cd(II) Transport in Seawater and Desalination Brines

**DOI:** 10.3390/polym18151854

**Published:** 2026-07-29

**Authors:** Nasim Khatir, Magdalena Cifuentes-Cabezas, Enriqueta Anticó, Clàudia Fontàs

**Affiliations:** Chemistry Department, University of Girona, C/Maria Aurelia Capmany, 69, 17003 Girona, Spain; nasim.khatir1986@gmail.com (N.K.); magdalena.cifuentes@udg.edu (M.C.-C.); enriqueta.antico@udg.edu (E.A.)

**Keywords:** polymer inclusion membranes, polymer, plasticizer, cadmium(II), sea water, desalination brine, improved membrane design

## Abstract

The transport of metal ions from highly saline matrices remains challenging due to the elevated ionic strength and complex chemical speciation that characterize seawater and desalination brines. In this work, the influence of membrane composition on Cd(II) transport through polymer inclusion membranes (PIMs) was evaluated using cellulose triacetate (CTA), poly(vinyl chloride) (PVC), and poly(vinylidene fluoride-co-hexafluoropropylene) (PVDF-HFP) membranes containing Aliquat 336. Membranes were tested using model NaCl solutions, seawater, and desalination brine, and the effects of membrane mass, polymer matrix, carrier/plasticizer composition, and receiving phase were investigated. Reducing the CTA membrane mass by 50% did not significantly affect transport efficiency, indicating that membrane composition had a greater influence on Cd(II) transport than membrane mass under the conditions evaluated. Among the evaluated formulations, the optimum PVDF-HFP membrane contained 60 wt.% PVDF-HFP, 30 wt.% Aliquat 336, and 10 wt.% butyl stearate (BTS), achieving a transport efficiency of 95.5% and an initial flux of 2.7 × 10^−6^ mol m^−2^ s^−1^ in desalination brine. The use of 0.5 M HNO_3_ as the receiving phase markedly improved Cd(II) transport in both synthetic saline solutions and real seawater and desalination brine compared with ultrapure water. These results highlight the importance of polymer–plasticizer interactions in controlling Cd(II) transport and demonstrate the potential of PVDF-HFP/Aliquat 336/BTS membranes for metal recovery from complex saline media.

## 1. Introduction

Polymer inclusion membranes (PIMs) have emerged as versatile materials for selective ion separation, extraction, and transport. A typical PIM consists of a polymer matrix that provides mechanical support, a carrier responsible for selective extraction and transport of the target species, and, in many cases, a plasticizer that facilitates diffusion within the membrane phase. Compared with conventional liquid membranes, PIMs offer improved stability, reduced carrier loss, and simplified operation, making them attractive systems for metal separation, recovery, and analytical preconcentration [1,2,3].

The transport performance of PIMs is strongly dependent on membrane composition. While the carrier determines extraction selectivity, the polymer matrix and plasticizer influence membrane structure, carrier mobility, and mass-transfer kinetics [3,4]. Consequently, membrane composition remains one of the key factors governing PIM performance. Recent reviews have highlighted that relatively small changes in polymer–plasticizer interactions can significantly modify membrane properties and transport behaviour, emphasizing the need for systematic studies evaluating the contribution of individual membrane components [5,6,7].

Cellulose triacetate (CTA) and poly(vinyl chloride) (PVC) remain the most widely employed polymers in PIM fabrication owing to their excellent film-forming properties and compatibility with a broad range of carriers and plasticizers [8,9,10]. Literature examples further illustrate the successful application of both polymers. For instance, a CTA-based PIM containing 1-alkylimidazole as a carrier achieved an initial Zn(II) transport flux (J_0_) of 2.65 µmol m^−2^ s^−1^ for the selective separation of Zn(II) from Mn(II) [11], while plasticizer-free formulations consisting of 50 wt.% CTA and 50 wt.% Aliquat 336 quantitatively separated toxic arsenic oxyanions within 5 h [12]. Likewise, PVC-based PIMs have shown excellent transport performance. For example, a membrane containing 55 wt.% PVC and 45 wt.% D2EHPA achieved an initial Zn(II) flux of 2.58 µmol m^−2^ s^−1^ [13], whereas a plasticizer-free membrane composed of 60 wt.% PVC and 40 wt.% Aliquat 336 successfully removed Cr(VI) from aqueous solutions within 3 h [14]. However, the search for alternative polymer matrices has intensified in recent years, motivated by the need to improve membrane stability, expand fabrication possibilities, and enhance performance under demanding operating conditions. Among the available alternatives, poly(vinylidene fluoride-co-hexafluoropropylene) (PVDF-HFP) has attracted growing interest due to its excellent chemical resistance, mechanical robustness, and compatibility with a wide range of extractants and ionic liquids (ILs), such as Aliquat 336, D2EHPA or trioctyl(dodecyl)phosphonium chloride [15,16,17]. Previous studies have demonstrated the successful application of PVDF-HFP-based PIMs for the extraction and transport of Cu(II), V(V), As(V), Cr(VI), Rh(III), Au(III), Bi(III), and thiocyanate species [17,18,19,20,21,22]. Despite these promising results, information regarding Cd(II) transport through PVDF-HFP-based PIMs remains scarce, and systematic comparisons with conventional CTA- and PVC-based membranes under identical operating conditions are largely unavailable.

Besides the polymer matrix, membrane performance is also strongly influenced by the carrier and plasticizer. Among the carriers employed in PIMs, quaternary ammonium salts such as Aliquat 336 (trioctylmethylammonium chloride) are particularly attractive because of their strong affinity for anionic metal complexes [8,23]. Aliquat 336 acts as the mobile carrier (extractant) in the membrane phase, selectively extracting metal complexes at the feed/membrane interface, transporting them across the membrane, and releasing them into the receiving phase. Owing to this transport mechanism, it has been widely employed in PIMs for the selective separation of metal ions, including Cd(II), Au(III), and V(V) [9,20,21].

In addition to its role as an anion exchanger, Aliquat 336 can also contribute to membrane plasticization by increasing flexibility and facilitating ion mobility within the membrane phase [8,24,25]. Nevertheless, despite this partial plasticizing contribution, additional plasticizers are generally required to achieve efficient transport performance and adequate membrane stability. Conventional plasticizers such as 2-nitrophenyl octyl ether (NPOE), dibutyl phthalate (DBP), and dioctyl phthalate (DOP) are widely employed because they enhance carrier mobility, reduce diffusional resistance, and modify membrane microstructure, directly affecting transport behavior [4,26,27]. More recently, butyl stearate (BTS) has emerged as a promising alternative plasticizer capable of providing membrane characteristics and transport performances comparable to conventional systems while maintaining excellent membrane stability [26]. However, information regarding its behavior in Cd(II)-selective PIMs and its interaction with different polymer matrices remains limited.

Cadmium was selected as the target metal due to its environmental relevance and its characteristic speciation behavior in chloride-rich media. In highly saline solutions, Cd(II) forms stable chloro-complexes, including CdCl_3_^−^ and CdCl_4_^2−^, whose distribution strongly depends on chloride concentration [23,28]. The formation of these anionic species favours extraction by anion-exchange carriers such as Aliquat 336, making Cd(II) an excellent model system for investigating carrier-mediated transport through PIMs. Several studies have demonstrated the successful transport of Cd(II) through CTA- and PVC-based membranes containing Aliquat 336 and conventional plasticizers, highlighting the critical influence of membrane formulation on transport efficiency [9,10,29]. More recently, Cd(II) transport and preconcentration have also been demonstrated in seawater using PIM-based devices, confirming the applicability of this approach in saline environments [30,31]. Nevertheless, most studies have focused on conventional membrane formulations and relatively limited membrane compositions.

Moreover, the interest in Cd(II) transport under saline conditions is relevant in the broader context of metal recovery from complex aqueous matrices. Seawater desalination is generating increasing volumes of concentrated brines worldwide, which are increasingly being regarded as secondary resources rather than waste streams [32,33,34]. These highly saline matrices contain significant concentrations of salts and trace metals that may potentially be recovered as valuable resources [35,36]. However, their elevated ionic strength and complex composition represent major challenges for conventional separation technologies [37,38]. In this context, emerging membrane-based approaches for brine concentration and minimal or zero liquid discharge are receiving increasing attention [39]. In chloride-rich environments, Cd(II) speciation is strongly influenced by chloride concentration, directly affecting extraction equilibria and membrane transport [23]. Consequently, understanding the influence of membrane composition on Cd(II) transport under highly saline conditions is essential for the development of robust membrane systems capable of operating in real seawater and brine matrices.

Despite the extensive literature on PIMs, systematic studies evaluating the combined influence of the polymer matrix and plasticizer composition on Cd(II) transport under saline conditions remain scarce. In particular, direct comparisons of CTA-, PVC-, and PVDF-HFP-based membranes under identical experimental conditions are lacking, making it difficult to distinguish the specific contribution of the polymer matrix to membrane performance. Furthermore, the potential of BTS as a more sustainable alternative to conventional plasticizers for Cd(II) transport and the applicability of PVDF-HFP-based membranes in highly saline matrices have not been thoroughly investigated.

Therefore, the objective of this work was to systematically evaluate the influence of polymer matrix and plasticizer composition on Cd(II) transport through Aliquat 336-based PIMs. Membranes based on CTA, PVC, and PVDF-HFP were prepared using NPOE and BTS as plasticizers and evaluated under saline conditions. By directly comparing these membrane systems under identical operating conditions, this study provides new insights into the role of polymer–plasticizer interactions in governing membrane performance. Particular attention was devoted to understanding how polymer–plasticizer interactions affect membrane performance and to assessing the suitability of PVDF-HFP as an alternative membrane matrix. Finally, the selected membrane system was evaluated using synthetic saline solutions, seawater, and desalination brine to validate its applicability under realistic high-salinity conditions.

## 2. Materials and Methods

### 2.1. Reagents and Solutions

CTA (Acros Organics, Geel, Belgium), PVC and PVDF-HFP, both from Sigma-Aldrich (Taufkirchen, Germany), were used as base polymers for the preparation of PIMs. Aliquat 336 (Sigma-Aldrich, St. Louis, MO, USA) was employed as the carrier, while NPOE (Sigma-Aldrich, Buchs, Switzerland) and BTS (Sigma-Aldrich, Saint-Quentin-Fallavier, France) were used as plasticizers. Different casting solvents were selected depending on the polymer: chloroform (Sigma-Aldrich, Taufkirchen, Germany) for CTA-based PIMs, tetrahydrofuran (THF, PanReac-AppliChem, Darmstadt, Germany) for PVC-based PIMs, and acetone (Fisher Chemical, Saint-Josse-ten-Noode, Belgium) for PVDF-HFP-based PIMs.

Feed solutions consisted of 1 mg L^−1^ Cd(II) prepared in NaCl media at concentrations of 0.1, 0.5, and 3.4 M. The 0.5 M solution was used as a simplified synthetic sea-water according to standard marine chemistry values, whereas 3.4 M NaCl simulated desalination brine under zero-liquid discharge (ZLD) treatment conditions according to industrial literature values [30,34]. Solutions were prepared by dilution of a 1000 mg L^−1^ Cd(II) standard solution (Merck, Darmstadt, Germany) using sodium chloride (NaCl, PanReac-AppliChem, Barcelona, Spain).

Seawater samples were collected from the Mediterranean Sea (Palamós, Girona, Spain), and brine samples were obtained from a seawater desalination plant located in Tordera (ITAM, Catalonia, Spain). The desalination facility operates by reverse osmosis, producing a concentrated brine stream as a by-product. The main physicochemical characteristics of the samples are summarized in Table 1. Ultrapure water (Milli-Q Plus water purification system, Millipore Iberica S.A., Madrid, Spain) was used as the receiving phase in all experiments. Nitric acid (HNO_3_) was used when required.

### 2.2. PIM Preparation

PIMs were prepared using a conventional solvent casting–evaporation method. The experimental design followed in this work was based on the progressive modification of conventional PIM formulations in order to evaluate the influence of membrane composition on Cd(II) transport under highly saline conditions. In this context, different membrane design elements were considered, including the reduction in membrane mass, the replacement of NPOE by BTS as plasticizer, and the use of acetone as casting solvent for PVDF-HFP-based membranes. Membrane mass reduction was based on recent studies demonstrating the feasibility of low-mass PIM configurations without compromising performance [35], while BTS was selected as an alternative plasticizer due to its favourable membrane properties [22]. The incorporation of PVDF-HFP and acetone was motivated by recent efforts to develop alternative membrane fabrication systems using less hazardous solvents [11,36]. These strategies are summarized in Table 2.

A first set of membranes was prepared to evaluate the effect of membrane mass, polymer matrix and plasticizer type. For these membranes, the polymer/extractant/plasticizer mass ratio was kept constant at 30/30/40 wt.%, using Aliquat 336 as the extractant and either NPOE or BTS as the plasticizer. This composition was selected based on previous studies showing that the presence of a plasticizer is essential for Cd(II) transport, with optimal performance previously reported for CTA-based PIMs plasticized with NPOE [9]. The compositions of this first set of membranes are reported in Table 3.

The polymer was dissolved in the corresponding solvent (chloroform for CTA, THF for PVC, and acetone for PVDF-HFP) under stirring at 300 rpm. For CTA, stirring was performed at 35 °C, while for PVC and PVDF-HFP it was carried out at room temperature. After complete dissolution, the carrier (Aliquat 336) and plasticizer were added, and the mixture was further stirred for 1 h to ensure homogeneity. The resulting solution was poured into a glass Petri dish (9.0 cm diameter), loosely covered, and left undisturbed at room temperature for 24 h to allow complete solvent evaporation.

A second set of PVDF-HFP/Aliquat 336/BTS membranes was prepared to evaluate the effect of liquid-phase content on Cd(II) transport. In this case, the Aliquat 336 content was kept constant at 30 wt.%, while the BTS content was varied from 40 to 10 wt.%. Consequently, the PVDF-HFP fraction was adjusted from 30 to 60 wt.%. The liquid phase (LP) was defined as the sum of Aliquat 336 and BTS. The compositions of these membranes are shown in Table 4.

### 2.3. PIM Preconcentration Experiments

Transport experiments to evaluate the effect of membrane mass, polymer matrix and plasticizer type were carried out using a PIM-based device designed for simultaneous extraction and preconcentration. A detailed description of the device has been reported elsewhere [22,40]. Briefly, 100 mL of the feed solution was placed in a glass beaker under constant magnetic stirring. The PIM device, with the membrane positioned at its base (membrane area = 1.78 cm^2^) and containing the receiving phase (ultrapure water or HNO_3_), was then immersed approximately 1 cm below the surface of the feed solution.

Experiments were conducted for 24 h at room temperature (22 ± 1 °C) using the set of membranes described in Table 3. After this period, the feed and receiving solutions were collected and analyzed. All experiments were performed at least in triplicate.

To evaluate membrane performance, the concentration of Cd(II) in the receiving phase was analyzed. Transport efficiency (TE) was calculated as:

(1) $$TE\left(\%\right)=\frac{{\left[Cd\right]}_{receiving,t}\times V_{receiving}}{{\left[Cd\right]}_{receiving,t}\times V_{feed}}\times 100$$

where [*Cd*] represents the Cd(II) (mg L^−1^) concentration and *V_feed_* and *V_receiving_* correspond to the volumes of the feed and receiving phases, respectively.

### 2.4. Two-Compartment Cell Transport Studies

To specifically investigate the effect of the liquid phase content on transport kinetics, experiments were conducted using a two-compartment transport cell and using the PIMs made of PVDF-HFP/Aliquat 336/BTS with compositions shown in Table 4. This setup consisted of two identical chambers separated by the PIMs (mmbrane area = 11.5 cm^2^) [41]. The feed compartment was filled with 200 mL of a saline solution (seawater or desalination brine) with 5 mg L^−1^ of Cd(II), while the receiving compartment contained 200 mL of 0.5 M HNO_3_.

Both compartments were continuously stirred at 400 rpm at room temperature. To monitor the transport kinetics, aliquots were periodically collected from both the feed and receiving solutions at predetermined time intervals up to 5 h. Transport efficiency (TE) was calculated according to Equation (1). The initial transport flux (*J*) was determined from the linear region of the Cd(II) accumulation profile in the receiving phase over time, according to:

(2) $$J=\frac{\;V_{receiving}}{S}\left(\frac{dC}{dt}\right)$$

where *S* is the exposed membrane area and *dC*/*dt* corresponds to the slope of the linear variation in Cd(II) concentration as a function of time.

### 2.5. PIM Leaching Studies

Leaching experiments were performed on the selected PVDF-400-BTS membrane following the gravimetric approach commonly used for Aliquat 336-based PIMs [42]. Membrane samples (1.5 × 1.5 cm) were separately immersed in 25 mL of 0.5 M NaCl, seawater, or desalination brine under continuous orbital stirring for 24 h at room temperature (22 ± 1 °C). Each membrane sample was weighed before immersion and again after the leaching experiment, following drying to constant weight. Leaching was evaluated as the percentage of membrane mass loss after exposure to each solution [43].

### 2.6. Instruments

Metal concentrations were determined by microwave plasma–atomic emission spectroscopy (MP-AES 4200, Agilent Technologies, Tokyo, Japan).

Membrane morphology was examined by field emission scanning electron microscopy (FE-SEM; S-4100, Hitachi, Tokyo, Japan). Prior to analysis, membrane samples were mounted on aluminium stubs and carbon-coated using a turbo carbon evaporator (Model K950, Emitech, Montigny-le-Bretonneux, France). Image acquisition and processing were carried out using Quartz PCI software (Vancouver, BC, Canada).

The thermal stability of the selected PVDF-HFP-based PIMs and their individual components was evaluated by thermogravimetric analysis (TGA) using a Mettler Toledo TGA/DSC combined instrument (Mettler Toledo, L’Hospitalet de Llobregat, Barcelona, Spain). Analyses were performed from 30 to 650 °C at a heating rate of 10 °C min^−1^ under a nitrogen atmosphere (40 mL min^−1^).

## 3. Results and Discussion

### 3.1. PIM Formation and Morphological Characterization

**Figure 1:**
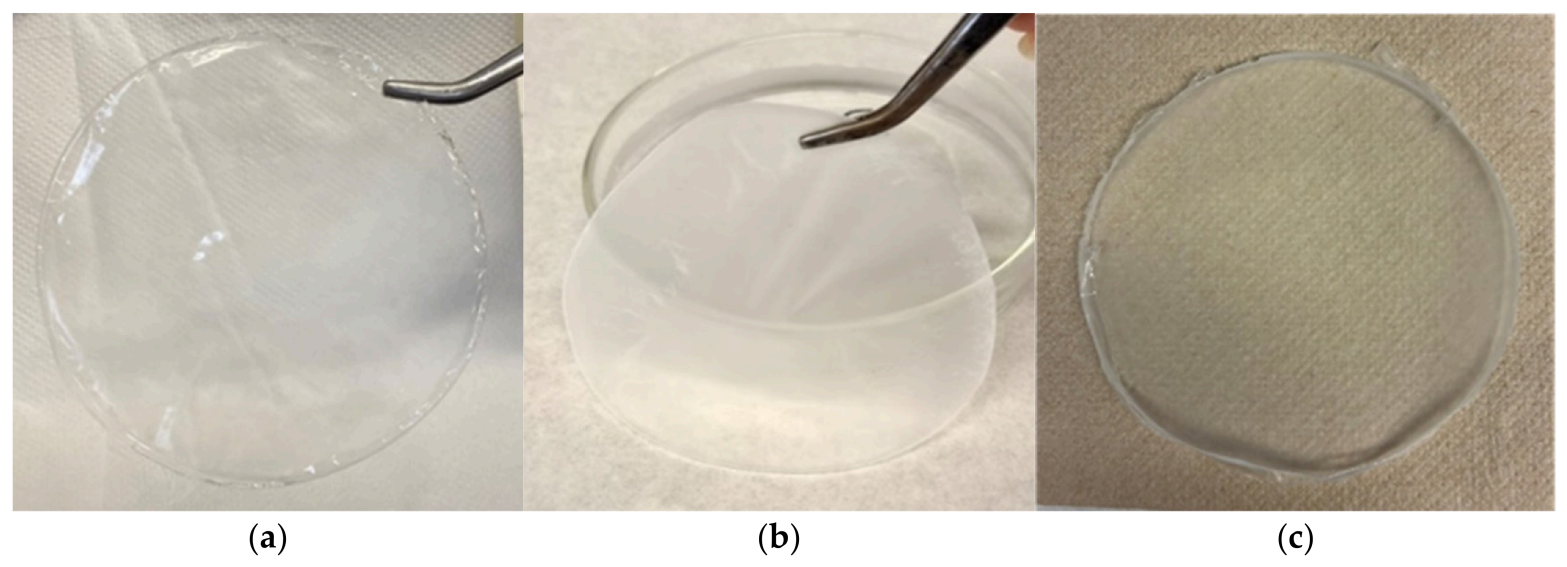
Visual appearance of the prepared PIMs. (**a**) CTA-based PIMs prepared with 100 mg of polymer, (**b**) PVDF-HFP-based PIMs prepared with 200 mg of polymer, (**c**) PVDF-HFP prepared with 400 mg.

The successful formation of the different membranes strongly depended on both the polymer type and the amount employed. For CTA-based PIMs, homogeneous, transparent, and mechanically stable films were successfully obtained using both the conventional polymer amount (200 mg) and a reduced amount of 100 mg (Figure 1a), confirming that the amount of polymer can be significantly reduced without compromising membrane integrity.

In the case of PVC-based PIMs, the polymer content could be reduced from the conventional 400 mg to 200 mg while still obtaining homogeneous and mechanically stable membranes. However, further reduction to 100 mg did not allow proper membrane formation, as the resulting films were excessively thin and adhesive, preventing the formation of flat self-supported membranes.

For PVDF-HFP-based PIMs, the main limitation was related to mechanical resistance rather than film formation. Membranes prepared using low polymer amounts (100–200 mg) were fragile and broke easily during handling (Figure 1b). Therefore, the polymer amount was increased to 400 mg to ensure adequate mechanical stability for transport experiments (Figure 1c).

**Figure 2:**
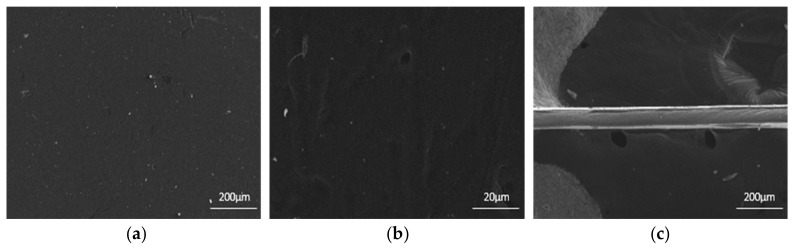
SEM images of CTA-100-BTS PIMs (30% CTA, 30% Aliquat 336, 40% BTS *w*/*w*): surface (**a**,**b**) and cross-section (**c**).

FE-SEM analysis was performed for selected membranes, namely CTA-100-BTS and PVDF-HFP-400-BTS, to evaluate their morphology. As shown in Figure 2, the CTA-based PIMs exhibit the typical morphology of conventional PIMs, characterized by a homogeneous and compact structure without visible pores. This observation confirms that the incorporation of BTS does not significantly alter the morphology of CTA-based PIMs. Similar dense and uniform structures are widely reported for PIM systems, where the carrier phase is immobilized within a compact polymer matrix [1,2].

**Figure 3:**
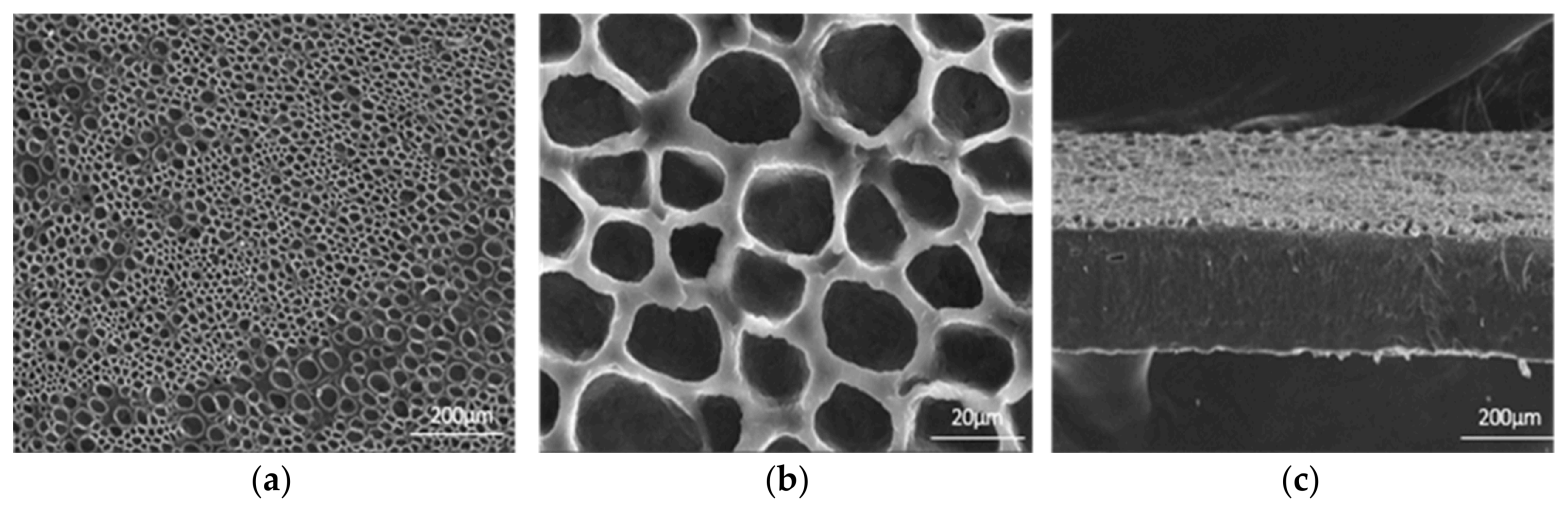
SEM images of PVDF-HFP-400-BTS PIMs (30% PVDF-HFP, 30% Aliquat 336, 40% BTS *w*/*w*): surface (**a**,**b**) and cross-section (**c**).

In contrast, the PVDF-HFP-based PIMs exhibited a different surface morphology, with pore-like features distributed across the membrane surface (Figure 3). At higher magnification, these features appeared as interconnected cavities or microvoids. However, cross-sectional images revealed that the membrane preserved a dense internal structure, similar to that observed for conventional PIMs. This indicates that the observed porosity is mainly a surface-related feature associated with the solvent evaporation process during membrane formation rather than the presence of a fully porous bulk structure. Similar morphological deviations, including the formation of microvoids and heterogeneous domains, have been reported in PIM systems when variations in membrane composition or preparation conditions affect the internal organization of the polymer matrix [3,4].

The observed morphology is consistent with previous studies on PVDF-HFP-based PIMs prepared by solvent casting, where solvent evaporation dynamics significantly influence membrane structure and porosity development [16,21]. Rapid evaporation of volatile solvents such as acetone may promote local phase separation phenomena and free-volume generation during membrane formation [16]. Despite the distinct surface features observed, the cross-sectional images confirm that the membrane behaves as a dense PIM, where transport occurs through diffusion of the metal–carrier complex within the polymeric phase rather than through continuous pores.

**Figure 4:**
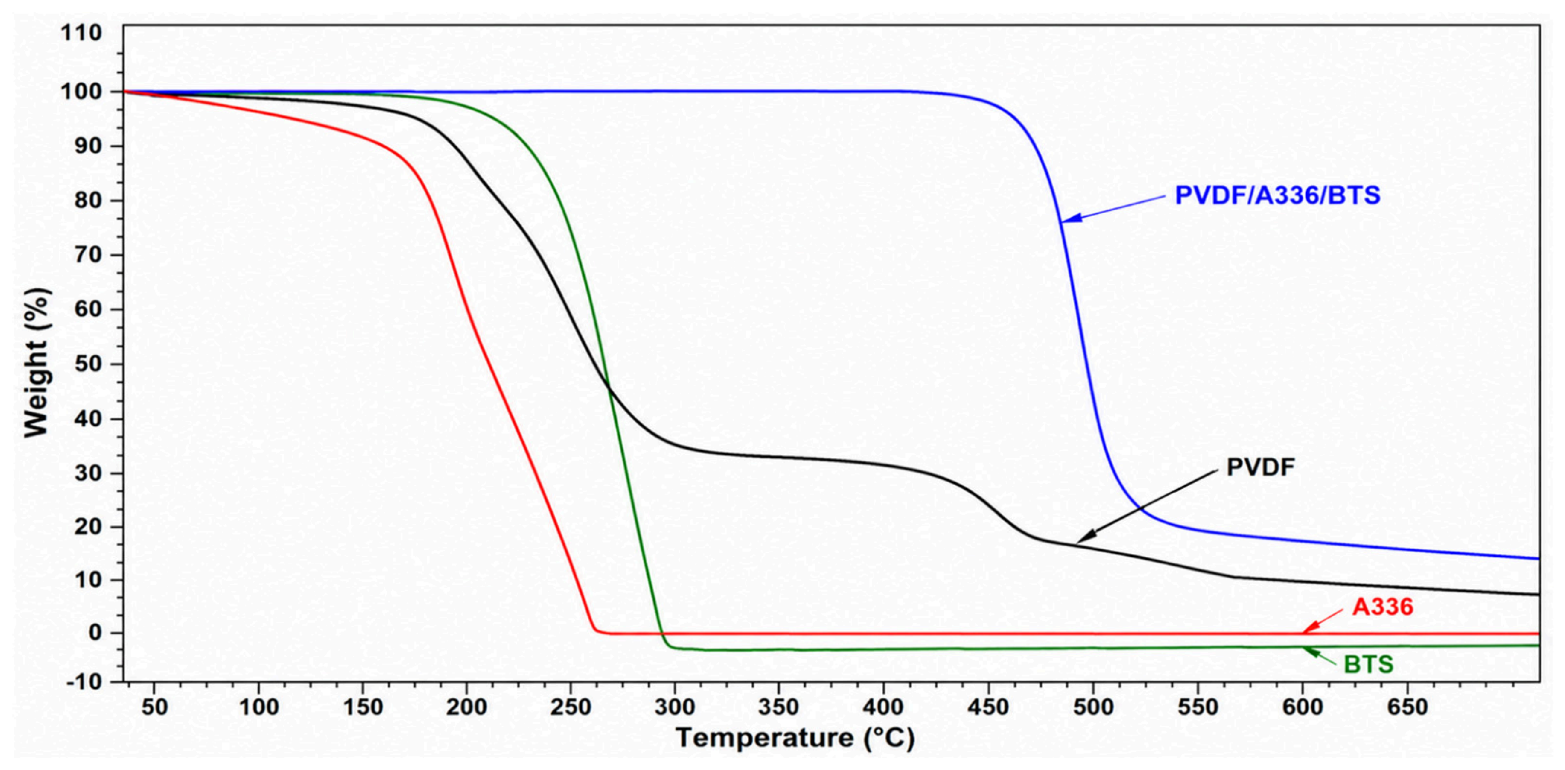
TGA curves of PVDF-HFP, Aliquat 336, BTS, and the corresponding PVDF-HFP/Aliquat 336/BTS PIMs.

The thermal stability of the selected PVDF-HFP-based PIMs was evaluated by TGA (Figure 4). As expected, PVDF-HFP exhibited the highest thermal stability, whereas BTS and Aliquat 336 showed degradation at lower temperatures. The membrane displayed a multistep degradation profile corresponding to the decomposition of its individual components, confirming the successful incorporation of the carrier and plasticizer within the polymer matrix. Importantly, all degradation events occurred at temperatures well above those used during membrane preparation and transport experiments, demonstrating the adequate thermal stability of the membrane system.

### 3.2. Effect of Polymer Matrix and Plasticizer on Cd(II) Transport

**Figure 5:**
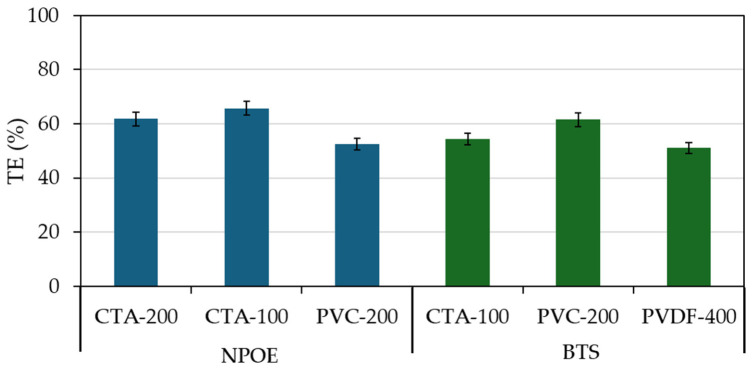
Cd(II) transport efficiency for different PIMs under model solution (0.1 M NaCl) with milli-Q water as receiving phase (*n* = 3).

The influence of polymer matrix and plasticizer composition on Cd(II) transport was evaluated using PIMs containing 30 wt.% polymer, 30 wt.% Aliquat 336, and 40 wt.% plasticizer (see Table 3). Transport experiments were performed using a model solution containing 0.1 M NaCl and Milli-Q water as the receiving phase. Results are shown in Figure 5. As it can be observed, all membrane formulations exhibited effective Cd(II) transport, with transport efficiencies ranging from approximately 51 to 66%, confirming the suitability of Aliquat 336-based PIMs for Cd(II) extraction and transport in chloride-containing media.

A comparison between CTA membranes prepared with 200 and 100 mg of polymer, corresponding to membrane thicknesses of 72 and 33 µm, respectively, showed only minor differences in transport efficiency. Despite the lower mass and thickness of the CTA-100-NPOE membrane, transport performance remained practically unchanged.

This behaviour indicates that Cd(II) transport is governed primarily by membrane composition and the physicochemical characteristics of the membrane phase rather than by the total amount of material employed. As long as membrane integrity is maintained, carrier-mediated transport remains effective. Similar observations have been reported for PIM systems, where mass transfer is mainly controlled by diffusion of the metal–carrier complex through the membrane phase rather than by membrane thickness alone [3,4]. Furthermore, recent studies demonstrated that reagent-efficient membrane configurations can substantially reduce material consumption without compromising transport performance when membrane homogeneity is preserved [43].

The effect of the plasticizer was strongly dependent on the polymer matrix. For CTA-based PIMs, replacing NPOE with BTS resulted in a slight decrease in transport efficiency, from approximately 66 to 54%. In contrast, PVC-based PIMs showed the opposite behavior, with transport efficiency increasing from approximately 52 to 61% when BTS was employed. These results demonstrate that the influence of a plasticizer cannot be considered independently of the polymer matrix and highlight the importance of polymer–plasticizer interactions in determining membrane performance.

Plasticizers constitute a quasi-liquid phase within PIMs and strongly influence carrier mobility, free volume, membrane flexibility, and diffusional resistance [3,4]. Consequently, transport efficiency results from a balance between stabilization of the extracted metal complex and its diffusional mobility through the membrane phase. NPOE exhibits a relatively high dielectric constant and viscosity, favouring stabilization of extracted species, whereas BTS is a less polar and less viscous aliphatic ester that may facilitate diffusion of the Cd–Aliquat 336 ion pair. Similar relationships between plasticizer properties, membrane microstructure, and transport performance have been widely reported for PIM systems [4,26,27]. In particular, Alcalde et al. [26] recently demonstrated that BTS can provide transport performances comparable to conventional plasticizers while maintaining favourable membrane characteristics.

Beyond the effect of the plasticizer, differences were also observed between the polymer matrices themselves. Even when maintaining identical carrier and plasticizer proportions, CTA- and PVC-based PIMs exhibited different transport efficiencies. Such behaviour is commonly reported in PIM systems and reflects differences in polymer polarity, chain mobility, free volume, and compatibility with both the carrier and the plasticizer [1,2,3]. The polymer matrix is not merely a mechanical support but actively contributes to the internal organization of the membrane phase and therefore influences the diffusion of metal–carrier complexes. Previous studies have shown that variations in polymer structure can significantly affect transport rates even when the same extractant is employed [3,10].

Among the investigated formulations, the PVDF-HFP-BTS membrane exhibited transport efficiencies comparable to those of PVC-based PIMs and only moderately lower than those obtained with CTA-based systems. This result is noteworthy considering the different physicochemical nature of PVDF-HFP and the distinct morphology observed by SEM (Figure 3). While the membrane surface exhibited microvoids and pore-like features, cross-sectional images confirmed the presence of a dense internal structure. Therefore, transport is still governed by diffusion of the Cd–carrier complex through the membrane phase rather than by convective flow through interconnected pores. This observation is consistent with the carrier-mediated transport mechanism generally described for PIMs [1,3].

The combination of satisfactory TE, adequate mechanical stability, and compatibility with acetone-based fabrication highlights the potential of PVDF-HFP as an alternative membrane matrix for Cd(II) transport. Although PVDF-HFP has previously been applied to the transport of Cu(II), V(V), Cr(VI), Rh(III), thiocyanate, and Bi(III) species [15,17,18,19,21,26], information regarding Cd(II) transport remains scarce. The present results therefore provide evidence that PVDF-HFP-based PIMs can operate efficiently in chloride-rich media and justify their selection for the subsequent evaluation under high-salinity conditions.

### 3.3. Performance of the Selected PVDF-HFP PIMs Under High-Salinity Conditions

**Figure 6:**
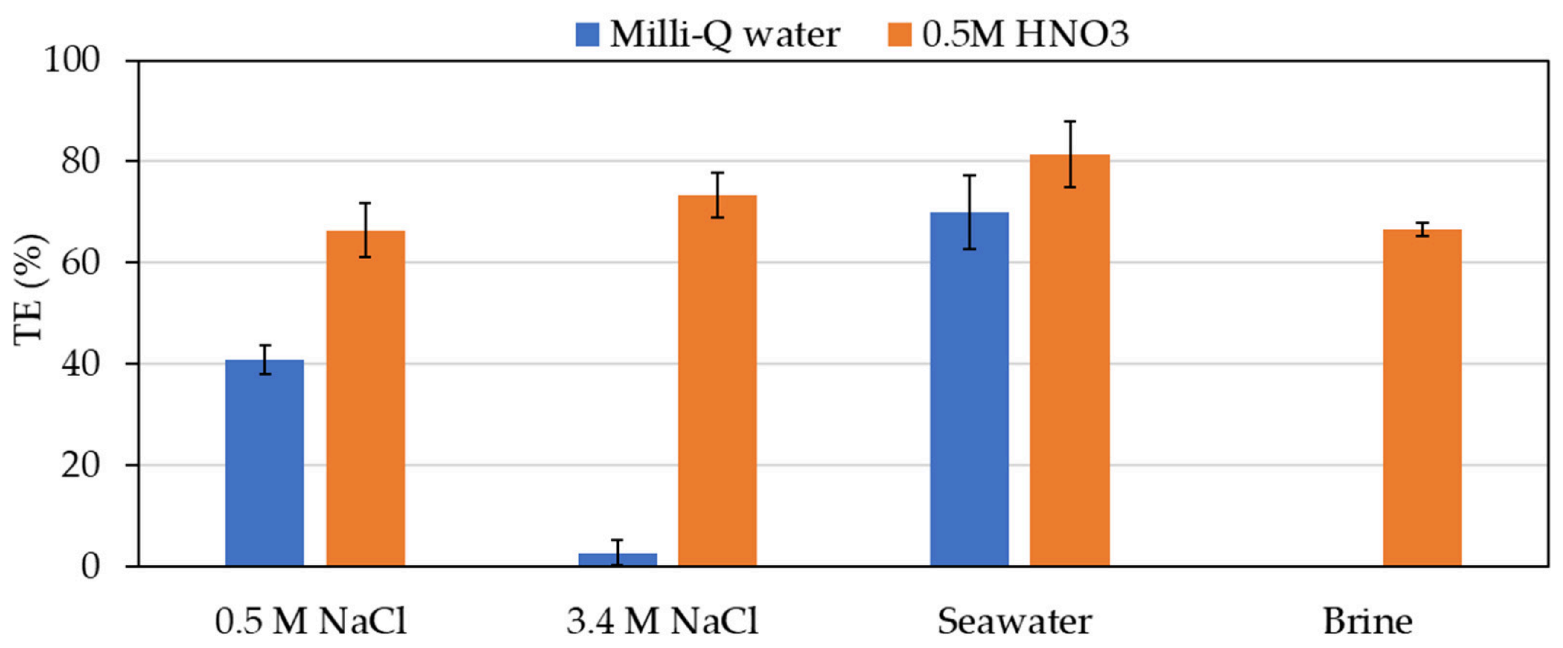
Effect of the receiving phase (ultrapure water vs. 0.5 M HNO_3_) on Cd(II) accumulation for PVDF-HFP-based PIMs under high-salinity conditions (*n* = 3).

The performance of the selected PVDF-HFP-BTS membrane was further evaluated under high-salinity conditions by comparing ultrapure water and 0.5 M HNO_3_ as receiving phases (Figure 6). Experiments were performed using both synthetic saline solutions (0.5 and 3.4 M NaCl) and real matrices, including seawater and desalination brine.

For all evaluated matrices, the use of 0.5 M HNO_3_ resulted in a marked increase in Cd(II) accumulation compared with ultrapure water, which became increasingly important as salinity increased. For the synthetic 0.5 M NaCl solution, transport efficiencies increased from approximately 40% with Milli-Q water to about 65% when 0.5 M HNO_3_ was employed. The effect was even more pronounced for the 3.4 M NaCl solution, where Cd(II) accumulation was almost negligible using ultrapure water, whereas efficient transport was restored in the presence of nitric acid, with transport efficiencies reaching approximately 70%. In seawater, transport efficiencies increased substantially when 0.5 M HNO_3_ was employed as the receiving phase, reaching values above 80%. For the desalination brine, Cd(II) accumulation was only observed when nitric acid was used, yielding transport efficiencies around 65%.

The enhanced performance observed with nitric acid can be attributed not only to the acidic conditions, which promote dissociation of the Cd–Aliquat 336 ion pair, but also to the participation of nitrate ions in carrier regeneration. The stripping process involves the release of Cd(II) into the receiving phase as Cd^2+^, a species that cannot be effectively re-extracted by the carrier. In addition, nitrate ions can interact with the quaternary ammonium groups of Aliquat 336 at the membrane/solution interface, facilitating carrier regeneration and thereby promoting the continuous transport of Cd(II) across the membrane [31].

To further evaluate the applicability of the system, transport experiments were performed using desalination brine samples containing different initial Cd(II) concentrations (0.1 and 1 mg L^−1^). Comparable transport efficiencies were obtained in both cases (64.0% and 66.6%), indicating that the transport process was not significantly affected by the initial Cd(II) concentration within the evaluated range. Under these conditions, Cd(II) concentrations in the receiving phase reached 1.28 ± 0.08 mg L^−1^ and 13.3 ± 0.3 mg L^−1^, respectively, confirming the capability of the system to effectively preconcentrate Cd(II) from highly saline matrices.

The ability of the PVDF-HFP membrane to maintain efficient Cd(II) transport in seawater and desalination brine demonstrates that the system is not significantly affected by the high ionic strength and complexity of these matrices. From an application perspective, membrane stability under saline conditions is also an important consideration. Therefore, the stability of the selected PVDF-HFP membrane was evaluated by gravimetric leaching experiments in 0.5 M NaCl, seawater and desalination brine. Low mass losses of 2.1, 2.4 and 2.7%, respectively, were obtained after 24 h of exposure, indicating good membrane stability under the investigated conditions.

These results are consistent with previous studies on Aliquat 336-based PIMs, which have shown that although Aliquat 336 may undergo noticeable leaching in ultrapure or deionized water, mass loss is markedly reduced in saline media. Kagaya et al. [44] reported significant mass loss for PVC-based Aliquat 336 PIMs in deionized water, whereas the presence of dissolved salts considerably suppressed membrane weight loss. Likewise, Dahdah et al. [45] demonstrated that CTA-based Aliquat 336 PIMs exhibited mass losses below 5 wt.% in concentrated nitrate and acidic saline media. Therefore, the low mass losses obtained in the present study are in good agreement with the reported stabilizing effect of saline solutions on Aliquat 336-containing PIMs.

Taken together, these findings are particularly relevant because seawater and desalination brines are increasingly recognized as secondary resources containing valuable elements, while their high salinity remains a major challenge for conventional separation technologies. The present results demonstrate that efficient Cd(II) transport can be achieved under realistic saline conditions using a simple functionalized polymeric membrane.

### 3.4. Effect of Liquid-Phase Content on Cd(II) Transport Kinetics

To further investigate the role of membrane composition on Cd(II) transport under realistic saline conditions, additional kinetic experiments were performed using PVDF-HFP-based PIMs with different liquid-phase contents. In these membranes, the Aliquat 336 content was kept constant at 30 wt.%, while the BTS content was varied and the PVDF-HFP fraction was adjusted accordingly, as reported in Table 4. As mentioned, the liquid phase (LP) was defined as the combined content of Aliquat 336 and BTS incorporated within the polymer matrix. This phase represents the functional domain where Cd(II) extraction and carrier-mediated diffusion occur. By decreasing the BTS content while maintaining the carrier concentration constant, the total LP content was reduced, allowing the influence of the immobilized liquid phase on Cd(II) transport behaviour to be assessed.

**Figure 7:**
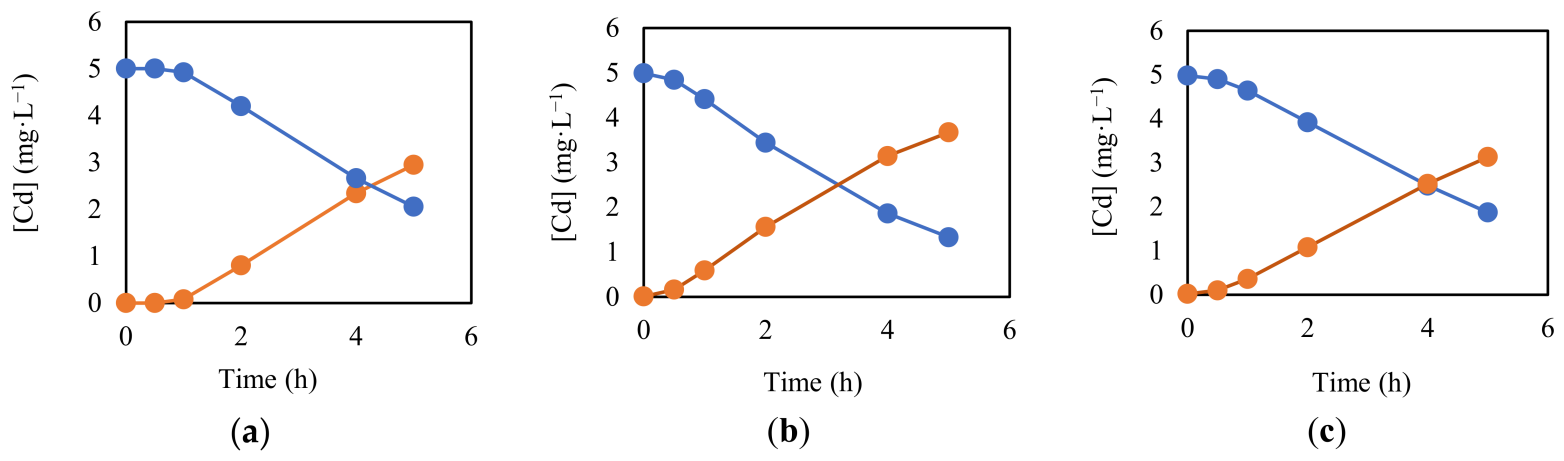
Effect of BTS content and total liquid-phase fraction on the transport kinetics of Cd(II) in seawater. (**a**) PVDF-HFP-70LP; (**b**) PVDF-HFP-60LP; (**c**) PVDF-HFP-40LP. • feed phase and • stripping phase.

**Figure 8:**
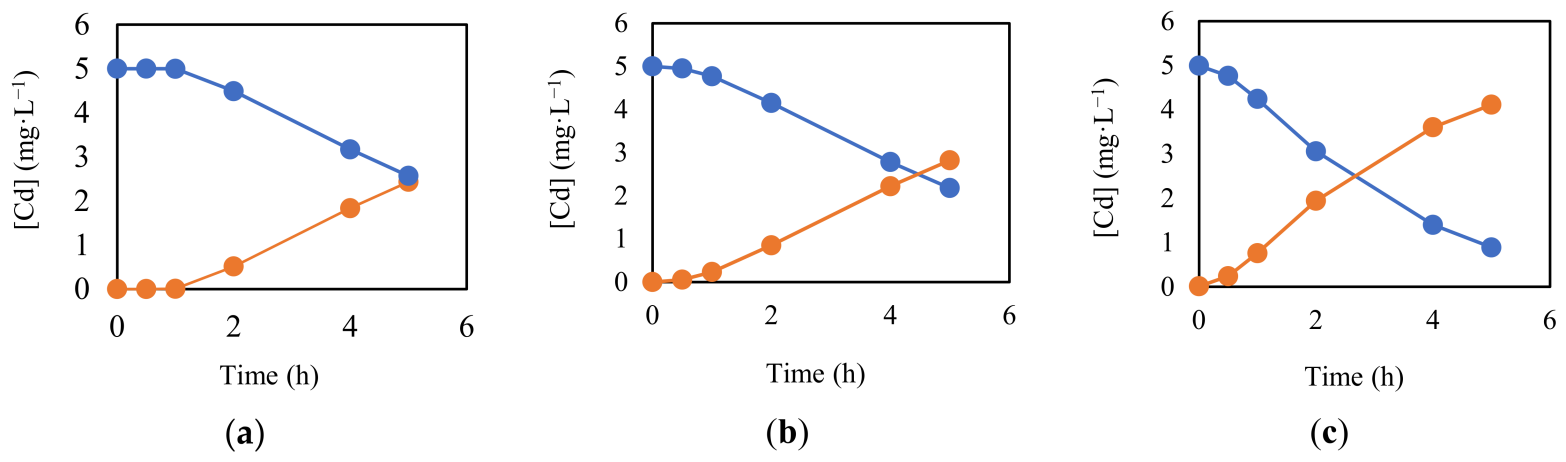
Effect of BTS content and total liquid-phase fraction on the transport kinetics of Cd(II) in desalination brine. (**a**) PVDF-HFP-70LP; (**b**) PVDF-HFP-60LP; (**c**) PVDF-HFP-40LP. • feed phase and • stripping phase.

Unlike the preconcentration experiments described above, these studies were carried out using a two-compartment transport cell, which allowed the simultaneous monitoring of Cd(II) concentrations in both the feed and receiving phases as a function of time. This configuration also enabled the evaluation of possible Cd(II) accumulation within the membrane phase during transport. Transport kinetics were investigated using both seawater and desalination brine as feed solutions, and the corresponding results are shown in Figure 7 and Figure 8, respectively.

As observed in Figure 7 and Figure 8, Cd(II) concentration in the feed phase progressively decreased in all experiments, while a corresponding increase was observed in the receiving phase. This confirms that Cd(II) was effectively transported across the membrane rather than only retained within it. The close correspondence between feed depletion and receiving-phase accumulation also suggests that no significant Cd(II) accumulation occurred within the membrane phase during the experiments.

Clear differences in transport kinetics were observed as a function of membrane composition. In general, membranes with lower BTS content showed equal or improved transport performance compared with the formulation containing the highest liquid-phase content. This behaviour was particularly evident in desalination brine, where the PVDF-HFP-40LP membrane enabled rapid Cd(II) depletion from the feed phase and effective transfer to the receiving phase within only a few hours. This result is especially remarkable considering the high ionic strength and chemical complexity of desalination brine, which are expected to hinder metal extraction and mass transfer.

Since the Aliquat 336 content was kept constant at 30 wt.% in all these membranes, the observed differences cannot be attributed to variations in the amount of carrier. Instead, they should be related to changes in the BTS/PVDF-HFP ratio and, consequently, to changes in the internal membrane environment. Aliquat 336 acts as the active carrier responsible for Cd(II) extraction and transport, whereas BTS contributes to the formation of a sufficiently mobile organic phase that facilitates diffusion of the carrier–metal ion pair through the membrane. However, increasing the BTS content does not necessarily improve transport. At fixed carrier content, an excessive amount of plasticizer may modify the organization of the membrane phase and generate less efficient transport pathways, while a higher PVDF-HFP fraction may improve membrane structural continuity and favour more efficient carrier-mediated transport under highly saline conditions.

The initial flux values reported in Table 5 further support this interpretation. In seawater, the highest flux was obtained for the PVDF-HFP-60LP membrane, while the PVDF-HFP-40LP membrane showed a flux comparable to that of PVDF-HFP-70LP. In desalination brine, however, reducing the liquid-phase content from 70LP to 40LP resulted in a marked increase in flux, from 12.1 to 27.0 × 10^−7^ mol m^−2^ s^−1^. These results indicate that the influence of liquid-phase content is matrix-dependent, but also confirm that a high plasticizer content is not required to achieve efficient Cd(II) transport. 

From a mechanistic perspective, this behaviour is consistent with the dual transport regimes described for PIMs. As reported by Rodríguez de San Miguel et al. [4], variations in the composition and distribution of the liquid phase can modify the dominant transport mechanism, shifting the system between carrier-diffusion and chained-carrier regimes. In the present study, the superior performance of the 40LP membrane suggests that reducing the BTS content favours a more efficient organization of the active liquid phase, improving carrier connectivity and reducing mass-transfer resistance. Similar behaviour has been described by percolation-based models, which predict the existence of an optimal organic-phase composition that maximizes transport without compromising carrier density [4,10].

Recent studies on alternative plasticizers further support this interpretation. For instance, Alcalde et al. [26] and Mahmoud et al. [27] reported that increasing plasticizer content beyond an optimal threshold leads to decreased TE, despite improved membrane flexibility, highlighting that transport in PIMs is governed by a complex interplay between viscosity, polarity, and microstructural organization rather than by a single physicochemical parameter.

Importantly, the fluxes obtained for the PVDF-HFP/Aliquat 336/BTS membranes remained within the same order of magnitude as those reported for conventional CTA/NPOE/Aliquat systems [9,26].

Overall, these results demonstrate that the liquid phase plays a central role in Cd(II) transport through PVDF-HFP-based PIMs. However, membrane performance is not governed only by the amount of liquid phase, but by the balance between carrier content, plasticizer content and polymer fraction. Appropriate optimization of this balance provides an effective strategy for enhancing Cd(II) transport kinetics in complex saline matrices.

## 4. Conclusions

This study demonstrates that the performance of Aliquat 336-based polymer inclusion membranes for Cd(II) transport under saline conditions is strongly governed by membrane composition. In CTA-based PIMs, reducing the membrane mass by 50% did not compromise Cd(II) transport efficiency, indicating that the amount of material used can be significantly decreased without negatively affecting membrane performance.

The replacement of NPOE by BTS showed that the effect of the plasticizer is closely linked to the polymer matrix. While CTA-based membranes containing BTS provided Cd(II) transport efficiencies comparable to those obtained with NPOE, improved performance was observed when BTS was combined with PVC and, especially, with PVDF-HFP. The PVDF-HFP/Aliquat 336/BTS system therefore proved to be a suitable alternative formulation, combining effective Cd(II) transport with the use of acetone as a less hazardous casting solvent.

The receiving phase was also found to be a critical parameter. Among the tested solutions, 0.5 M HNO_3_ provided the most effective stripping conditions, favouring Cd(II) transport in synthetic saline solutions as well as in real seawater and desalination brine.

Kinetic experiments with PVDF-HFP-based PIMs further showed that the liquid-phase content can be optimized to enhance Cd(II) transport. At a fixed Aliquat 336 content of 30 wt.%, reducing the BTS content and increasing the PVDF-HFP fraction improved transport under highly saline conditions. This effect was particularly evident in desalination brine, where the Cd(II) flux increased from 12.1 × 10^−7^ mol m^−2^ s^−1^ for the PVDF-HFP-70LP membrane to 27.0 × 10^−7^ mol m^−2^ s^−1^ for the PVDF-HFP-40LP membrane.

Overall, these results demonstrate that efficient Cd(II) transport from complex saline matrices can be achieved by carefully balancing the polymer matrix, carrier, plasticizer, and receiving phase. The optimized PVDF-HFP/Aliquat 336/BTS PIMs represent a promising membrane platform for the selective recovery of Cd(II) from seawater and desalination brines, supporting future applications in sustainable desalination brine treatment, resource recovery, and circular water management.

## Figures and Tables

**Table 1:** Physicochemical characteristics of the seawater sample and brine solution.

	pH	Conductivity (µS cm^−1^)	Cl^−^ (mg L^−1^)/(mols L^−1^)	SO_4_^2−^ (mg L^−1^)	Na^+^ (mg L^−1^)	Ca^2+^ (mg L^−1^)
**Seawater**	8.10	66,100	21,075/0.59	2987	10,750	442.95
**Brine**	7.45	91,500	39,466/1.11	1882	22,490	847

**Table 2:** Membrane design elements considered in the preparation of PIMs for Cd(II) transport.

Aspect Evaluated	Initial/Conventional Approach	Alternative Approach Explored	Main Purpose/Benefit
Membrane mass	CTA-based PIMs with 200 mg polymer	CTA-based PIMs with 100 mg polymer	Reduction in membrane material by 50%
Plasticizer	NPOE	BTS	Reduction in solvent hazard
Casting solvent	THF/Chloroform	Acetone for PVDF-HFP	Lower solvent hazard

**Table 3:** Composition of the PIMs used to evaluate the effect of membrane mass, polymer matrix and plasticizer type. For these membranes, the polymer/Aliquat 336/plasticizer mass ratio was kept constant at 30/30/40 wt.%.

PIMs (30% Polymer/ 30% Extractant/40% Plastizicer)	Polymer (mg)	Extractant (mg)	Plasticizer (mg)	Thickness (µm)
CTA-200-NPOE	CTA (200)	Aliquat 336 (200)	NPOE (267)	72 ± 6
CTA-100-NPOE	CTA (100)	Aliquat 336 (100)	NPOE (133)	33 ± 3
CTA-100-BTS	CTA (100)	Aliquat 336 (100)	BTS (133)	47 ± 3
PVC-200-NPOE	PVC (200)	Aliquat 336 (200)	NPOE (267)	100 ± 14
PVC-200-BTS	PVC (200)	Aliquat 336 (200)	BTS (267)	91 ± 7
PVDF-400-BTS	PVDF-HFP (400)	Aliquat 336 (400)	BTS (533)	168 ± 10

**Table 4:** Composition of PVDF-HFP/Aliquat 336/BTS PIMs prepared with different liquid-phase contents.

PIMs	PVDF-HFP (wt.%)	Aliquat 336 (wt.%)	BTS (wt.%)	Liquid Phase (wt.%)
PVDF-HFP-70LP	30	30	40	70
PVDF-HFP-60LP	40	30	30	60
PVDF-HFP-40LP	60	30	10	40

**Table 5:** Effect of BTS content and total liquid-phase fraction on Cd(II) flux in seawater and desalination brine.

Feed Solution	PIM	Liquid Phase	J (×10^−7^ mol m^−2^ s^−1^)
Sea water	PVDF-HFP-70LP	70%	15.5
	PVDF-HFP-60LP	60%	20.0
	PVDF-HFP-40LP	40%	15.8
Desalination brine	PVDF-HFP-70LP	70%	12.1
	PVDF-HFP-60LP	60%	16.2
	PVDF-HFP-40LP	40%	27.0

## Data Availability

Data are contained within the article.

## Abbreviations

The following abbreviations are used in this manuscript:

PIMs	Polymer Inclusion Membranes
NPOE	2-nitrophenyl octyl ether
BTS	butyl stearate
PVDF-HFP	poly(vinylidene fluoride-co-hexafluoropropylene)
CTA	Cellulose triacetate
PVC	Poly(vinyl chloride)
THF	Tetrahydrofuran
TE	Transport efficiency
J	Transport flux
